# Worse Health Status and Higher Incidence of Health Disorders in Rhesus Negative Subjects

**DOI:** 10.1371/journal.pone.0141362

**Published:** 2015-10-23

**Authors:** Jaroslav Flegr, Rudolf Hoffmann, Mike Dammann

**Affiliations:** 1 Division of Biology, Faculty of Science, Charles University in Prague, Prague, Czech Republic; 2 Department of Hematology, Na Homolce Hospital, Prague, Czech Republic; University of Palermo, ITALY

## Abstract

Rhesus-positive and Rhesus-negative persons differ in the presence-absence of highly immunogenic RhD protein on the erythrocyte membrane. The biological function of the RhD molecule is unknown. Its structure suggests that the molecular complex with RhD protein transports NH_3_ or CO_2_ molecules across the erythrocyte cell membrane. Some data indicate that RhD positive and RhD negative subjects differ in their tolerance to certain biological factors, including, *Toxoplasma* infection, aging and fatique. Present cross sectional study performed on 3,130 subjects) showed that Rhesus negative subjects differed in many indices of their health status, including incidences of many disorders. Rhesus negative subjects reported to have more frequent allergic, digestive, heart, hematological, immunity, mental health, and neurological problems. On the population level, a Rhesus-negativity-associated burden could be compensated for, for example, by the heterozygote advantage, but for Rhesus negative subjects this burden represents a serious problem.

## Introduction

Polymorphism in the Rhesus factor, namely the existence of a large deletion in the RHD gene [1] in a substantial fraction of the human population, has been an evolutionary enigma since the discovery of this factor in the 1930’s [2–5]. Theoretically, neither the RhD-negative allele can successfully spread in the RhD positive population nor the RhD-positive allele can spread in the RhD negative population [6,7]. Before the introduction of prophylactic treatment in 1968, a positive frequency dependent selection systematically penalized the less abundant allele because lots of children of RhD-negative women in the mostly RhD-positive population as well as children of RhD- positive men in the mostly RhD-positive population were dying of hemolytic anemia. It has been suggested that this polymorphism can be stabilized when the disadvantage of carriers of the locally rarer allele is counterbalanced by higher viability of their heterozygote children or by another form of frequency-dependent selection [6]. In the past seven years, several studies have demonstrated that Rhesus positive and Rhesus negative subjects differ in resistance to the adverse effects of parasitic infections, aging, fatigue and smoking [7–13]. A study performed on 250 blood donors has further shown that the resistance to effects of toxoplasmosis is higher in Rhesus positive heterozygotes than in Rhesus positive homozygotes and substantially higher than in Rhesus negative homozogotes [7]. This is the first direct evidence for the role of selection in favour of heterozygotes in stabilization of the RHD gene polymorphism in human populations. Such a mechanism is reminiscent of widely known situations with polymorphism in genes associated with sickle cell anaemia in geographic regions with endemic malaria [14].

The results of previous studies suggest that RhD negative homozygotes could have a worse health status than RhD positive population consisting of RhD positive homozygotes and heterozygotes. These results, however, were obtained on either rather small or rather specific populations, e.g. military personnel [13] or pregnant women [12]. To obtain more reliable data about situation in more typical populations, we run a large questionnaire study in a population of healthy Czech and Slovak volunteers. Using an electronic questionnaire distributed with a Facebook-based snowball method [15], we have screened a population of 3,130 subjects for indices of various health problems as well as for incidences of 225 diseases and disorders.

## Methods

### Ethics Statement

Only subjects older 18 years were invited and allowed to start the internet test. We erased data of 7 subjects who claimed to be younger as well as the data of all subjects who did not respond how old they were. The study, including the method of obtaining an electronic consent with a participation in the study (by pressing a particular button), was approved by the IRB of the Faculty of Science, Charles University (Komise pro práci s lidmi a lidským materiálem Přírodovědecké Fakulty Univerzity Karlovy)—No. 2014/21.

### Subjects

The subjects were invited to participate in the study using a Facebook-based snowball method [15] by posting an invitation to participate in “an experiment searching for associations between the blood group of a subject and his/her personality, performance, morphology and health” on the wall of the Facebook page “Guinea pigs” for Czech and Slovak nationals willing to take part in diverse evolutionary psychological experiments (www.facebook.com/pokusnikralici). The participants were informed about the aims of the study on the first page of the electronic questionnaire: “The subject of the present study is searching for associations between the blood group of a subject and his/her personality, performance, morphology and health. If you can check up on what your blood group is, please do it now. “They were also provided with the following information: “The questionnaire is anonymous and obtained data will be used exclusively for scientific purposes. Your cooperation in the project is voluntary and you can terminate it at any time by closing this web page. If you can check up on what your blood group is, please do it now. We need the data from subjects with all blood groups, not only from Rh negative subjects. Therefore, please share the link to this questionnaire with your friends, for example on Facebook. Press the “continue” button if you agree to your anonymous participation in the study”. The share button was pressed by 480 participants, which resulted in obtaining data from 4,286 responders in total between 28.4. 2014–9.3. 2015. Data file is available as the S1 File.

### Questionnaire

The anamnestic questionnaire was prepared by two medical doctors and was distributed as a Czech/English Qualtrics survey (http://1url.cz/q05K). It contained two categories of questions. The first of them monitored presence and intensity of general and specific health problems of responders. The responders were asked to subjectively rate of their allergic, cancer, digestive, fertility, genitourinary, heart, hematological, immunity, mental health, metabolic including endocrine, musculoskeletal, neurological, respiratory organs, sense organs, and sexual life problems using 6-points Likert.scales. The second group of questions tried to collect objective information reflecting the health status of responders. We asked the responders, for example, how many drugs prescribed by doctors they currently takes per day, how many of “different herbs, food supplements, multivitamins, superfoods etc.” they currently take per day, how many times they used antibiotics during the past 365 days. We also provided the responders lists of about 250 disorders (separated to 15 categories) and asked them to tick which of them they were diagnosed with. The questionnaire contained, among others, also the following questions: “What is your Rh blood group?” with three options: a) I do not know / I am not sure, b) negative (this is the less frequent variant) c) positive (the more frequent variant). Implicitly, the answer a) (I do not know/I am not sure) was checked.

### Statistical methods

Before statistical analysis, suspicious data (too high or too short body height, too low or too high body mass or age, too short duration of the test etc.) were filtered out (26 cases). In the test, we also measured simple reaction times, operational, short-term and long-term memory, psychomotor performance, intelligence and personality profiles. However, here we have analyzed only data concerning health status.

SPSS v. 21. was used for all statistical tests. Ordinal and binary data were analyzed by partial Kendall´s correlation test [16,17]. This test measures strength and significance of association between binary, ordinal and continuous data regardless of their distributions. This technique enabled us to control for one confounding variable, for example the age of a responder. The Excel sheet for computing partial Kendall’s *Tau* and the significance between variables A and B after the variable C is controlled based on Kendall *Tau*´s AB, AC and BC. It is available here: http://web.natur.cuni.cz/flegr/programy.php (item no. 12) and in S2 File. Certain diseases have very different incidence in men and women. Also, some biological factors, including RhD phenotype, could have different impacts on men and women. Therefore, we performed all analyses for all responders and also separately for the male and female responders.

## Results

### Descriptive statistics of data

**Fig 1:**
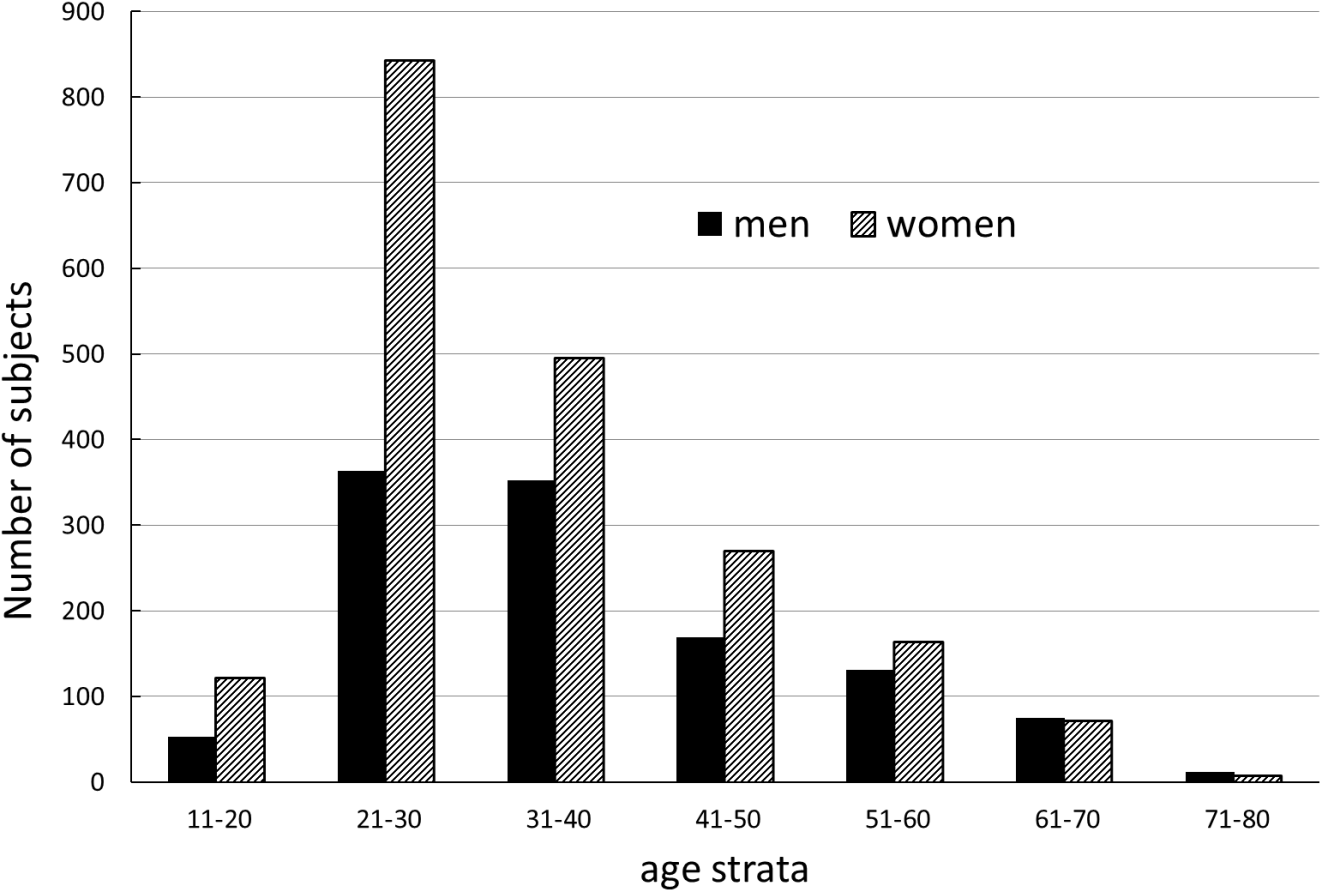
Age distribution for male and female participants of a study.

Among 4,286 Czech and Slovak participants of a subsequent case-control study, 3,130 subjects (840 RhD positive men, 317 RhD negative men, 1,337 RhD positive women and 636 RhD negative women) provided information about their gender and RhD phenotype. RhD negative subjects, especially women, have higher motivation to care about, and to remember, their RhD phenotype. Therefore, the frequency of RhD negative subjects (30.4%) differed from the 16% general frequencies within the Czech and Slovak populations and also between men (27.4%) and women (32.2%). The mean age of RhD positive men (37.6, *S*.*D*. 13.5) was approximately the same as that of RhD negative men (37.7, *S*.*D*. 12.7), *t*_*(1153)*_ = -0.10, *P* = 0.923. RhD positive women were younger (33.6, *S*.*D*. 11.9) than RhD negative women (35.2, *S*.*D*. 12.7), *t*_*(1923)*_ = 2.74, *P* = 0.006. The numbers of men and women in the particular age strata were comparable, with the exception of the 21–30 age stratum, which consisted of 363 men and 842 women (Fig 1).

### Correlation of RhD phenotype with self-reported health problems (ordinal variables)

Twenty-two dependent variables (mostly ratings of particular health problems on a scale from 1–6, 1: “no problems at all”, 6: “frequent or serious”) were ordinal and had a highly skewed distribution. Therefore, the nonparametric partial Kendall’s correlation test (which enables to control one confounding variable, here the age) was used to search for an association between the RhD phenotype and the intensity of fifteen categories of health problems (allergic, cancer, digestive, fertility, genitourinary, heart, hematological, immunity, mental health, metabolic including endocrine, musculoskeletal, neurological, respiratory organs, sense organs, and sexual life problems) and also another six health-related variables, namely the numbers of drugs prescribed by doctors that the subject currently takes per day, numbers of “different herbs, food supplements, multivitamins, superfoods etc.” the subject currently takes per day, how many times the subject has used antibiotics during the past 365 days, how many times the subject was required to seek acute medical care for a serious illness (not injury) that lasted more than 3 days during the past 5 years, how many specialized medical doctors the subject had to regularly attend (not for prevention) at least once in the past two years, how often the subject felt tired (not after exertion, e.g. sports) and how often the subject has experienced a headache. The results showed that the RhD negative subjects had more serious health problems in 6 of 22 analyzed variables than the RhD positive subjects (Table 1).

**Table 1:** Difference in various health status related variables between RhD negative and RhD positive subjects.

	all		men		women	
problems	*Tau*	*p*	*Tau*	*p*	*Tau*	*p*
allergic	0.018	0.153	0.016	0.444	0.012	0.450
cancer	0.008	0.514	-0.053	0.012*	0.031	**0.052**
digestive	0.034	0.008*	-0.016	0.443	0.050	0.002*
fertility	-0.009	0.475	0.045	0.035*	-0.042	0.009*
genitourinary	0.006	0.628	-0.026	0.223	-0.003	0.844
heart & vascular	0.031	0.015*	0.001	0.971	0.047	0.003*
hematological	0.028	0.028*	-0.028	0.185	0.031	**0.053**
immunity	0.034	0.007*	0.023	0.268	0.024	0.126
metabolic	-0.006	0.672	-0.020	0.342	-0.017	0.296
musculoskeletal	0.015	0.264	-0.039	**0.069**	0.035	0.034*
mental health	0.013	0.322	0.049	0.024*	-0.012	0.460
neurological	0.016	0.225	0.060	0.005*	-0.013	0.417
respiratory org.	-0.003	0.798	-0.002	0.918	-0.008	0.613
sense organs	-0.014	0.279	-0.033	0.120	-0.012	0.454
sexual life	-0.012	0.369	-0.011	0.615	-0.007	0.649
medicine/day	0.049	0.000*	0.047	0.025*	0.045	0.004*
herbs/day	-0.001	0.942	0.045	0.030*	-0.039	0.014*
antibiotics/year	0.014	0.269	-0.022	0.290	0.023	0.136
acute care/5 years	0.002	0.883	0.016	0.429	-0.013	0.418
doctors/2 years	0.018	0.162	-0.017	0.425	0.025	0.122
tired (frequency)	0.026	0.046*	0.010	0.637	0.019	0.238
headache (frequency)	0.013	0.321	0.024	0.264	-0.020	0.214

Number of responders varied between particular questions and was about 1,000 for men and 1,800 for women. Mostly significant effects of age on health status were controlled in present partial Kendall *Tau* test. Positivity of *Tau* indicates that RhD negative subjects have higher values of particular health related variables, i.e., a worse health status. Significant results (*P* < 0.05) and trends (*P* < 0.10) are printed in bold. Asterisks indicate results significant in two-sided tests. Values < 0.0005 are coded as 0.000.

### Association between RhD phenotype and incidence of particular diseases (binary variables)

After rating each particular category of health problems, the subjects were asked to identify which specific disorders they suffered from on the lists of 225 disorders. They were also asked to identify which specialized medical doctors they had to visit regularly (not for prevention) at least once in the past two years from a list of 10 types of specialists. The associations were analyzed with the partial Kendall’s *Tau* correlation test with age being a covariate. One hundred fifty four (154) of 225 diseases/disorders were reported by at least 10 subjects. Within this subset, 31 significant associations with RhD negativity (21 positive and 10 negative) were expressed in all subjects. In male subjects, the number of significant association was 35 (19 positive and 16 negative) while in female subjects the number of significant associations was 30 (18 positive and 12 negative). The expected number of false significant results for 462 statistical tests was not 96 but 23. For example, RhD negative men more often reported certain mental health disorders including panic disorders, antisocial personality disorders and attention deficits, ticks, fasciculation, thyroiditis, immunity disorders, allergies, especially skin allergies, excessive bleedings, anemia, osteoporosis, liver disease, infectious diseases and acute diarrhea diseases, while they less often reported gall bladder attacks, coeliac disease, maldigestion, malabsobtion, warts, some types of cancers and prostate hypertrophy. RhD negative women reported more frequently psoriasis, constipation and diarrheas, ischemic diseases, type 2 diabetes, some types of cancers, lymphatic nodes swelling, vitamin B deficiency, thrombosis, tonsil stones, too high sex desire, precocious puberty, urinary tract infections, scoliosis and they less often reported hearing loss, weight loss, hypoglycemia, glaucoma, fasciculation and warts. RhD negative subjects had to make more frequent visits to medical professionals specializing in otolaryngology (*P* = 0.021), psychiatry (*P* = 0.008), gynecology (*P* < 0.001), and dermatology (*P* = 0.014) (the theoretical number of false positive results was 0.5). Table 2 shows the associations between RhD negativity and disease incidences while Table 3 shows the associations between RhD negativity and visiting specialized doctors.

**Table 2:** Differences in incidences of particular disorders between RhD positive and RhD negative subjects.

	All						Men						Women					
	Rh-Dis-	Rh-Dis+	Rh+ Dis-	Rh+ Dis+	*Tau*	*P*	Rh-Dis-	Rh-Dis+	Rh+ Dis-	Rh+ Dis+	*Tau*	*P*	Rh-Dis-	Rh-Dis+	Rh+ Dis-	Rh+ Dis+	*Tau*	*P*
Pharyngitis	294	591	600	1379	-0.030	0.017*	100	185	255	498	-0.012	0.570	194	406	345	881	-0.045	0.004*
Bronchitis, pneumonia	686	199	1519	460	-0.009	0.476	233	52	602	151	-0.021	0.320	453	147	917	309	-0.009	0.558
Rhinitis, tonsilitis	367	518	847	1132	0.015	0.240	134	151	362	391	0.010	0.630	233	367	485	741	0.010	0.513
Ectoparasites, e.g. lice	738	147	1649	330	0.002	0.868	250	35	675	78	0.028	0.174	488	112	974	252	-0.018	0.253
Scabies	868	17	1930	49	-0.018	0.147	280	5	733	20	-0.026	0.203	588	12	1197	29	-0.014	0.378
Helminthiasis	844	41	1876	103	-0.012	0.325	272	13	723	30	0.013	0.545	572	28	1153	73	-0.027	**0.090**
Acute diarrhea dis.	724	161	1679	300	0.040	0.001*	238	47	658	95	0.051	0.014*	486	114	1021	205	0.031	0.046*
Acquired immunodef.	866	19	1935	44	-0.003	0.796	283	2	743	10	-0.026	0.202	583	17	1192	34	0.000	0.991
Flu and flu-like virosis	297	588	632	1347	-0.013	0.312	87	198	228	525	-0.001	0.945	210	390	404	822	-0.014	0.381
Borreliosis	801	84	1807	172	0.011	0.391	264	21	694	59	-0.009	0.677	537	63	1113	113	0.016	0.297
Other thick born dis	878	7	1962	17	-0.003	0.797	280	5	745	8	0.028	0.177	598	2	1217	9	-0.024	0.128
Sexually transmit. dis.	870	15	1934	45	-0.018	0.142	280	5	738	15	-0.008	0.712	590	10	1196	30	-0.024	0.118
Hepatitis A, E	878	7	1955	24	-0.022	**0.083**	283	2	741	12	-0.035	**0.088**	595	5	1214	12	-0.012	0.448
Hepatitis B	879	6	1970	9	0.013	0.303	282	3	748	5	0.019	0.355	597	3	1222	4	0.012	0.452
Herpes zoster	832	53	1855	124	-0.007	0.553	272	13	708	45	-0.028	0.181	560	40	1147	79	0.000	0.978
Herpes, oral or genital	623	262	1410	569	0.008	0.531	218	67	559	194	-0.023	0.259	405	195	851	375	0.017	0.275
Meningoencephalitis	872	13	1960	19	0.021	**0.087**	281	4	747	6	0.027	0.185	591	9	1213	13	0.017	0.279
Inflamm. of middle ear	618	267	1400	579	0.011	0.379	212	73	565	188	0.007	0.739	406	194	835	391	0.007	0.637
Eye infections	784	101	1739	240	-0.009	0.467	261	24	699	54	0.021	0.309	523	77	1040	186	-0.030	**0.058**
Other infectious dis.	823	62	1874	105	0.034	0.007*	265	20	719	34	0.050	0.015*	558	42	1155	71	0.024	0.131
Skin mycosis	686	199	1558	421	0.013	0.310	246	39	626	127	-0.040	**0.055**	440	160	932	294	0.028	**0.076**
Bacterial skin infect.	822	63	1851	128	0.013	0.285	265	20	704	49	0.009	0.654	557	43	1147	79	0.017	0.288
Warts	600	285	1244	735	-0.045	0.000*	194	91	479	274	-0.041	0.048*	406	194	765	461	-0.047	0.003*
Lymphatic modes swelling	840	45	1883	96	0.007	0.588	275	10	727	26	0.001	0.943	565	35	1156	70	0.006	0.712
Mononucleosis	778	107	1728	251	-0.007	0.587	262	23	690	63	-0.004	0.831	516	84	1038	188	-0.016	0.312
Tonsil stones	657	183	1534	330	0.047	0.000*	235	36	631	96	0.001	0.970	422	147	903	234	0.057	0.000*
Skin allergy	646	224	1482	462	0.022	**0.075**	230	52	633	104	0.054	0.010*	416	172	849	358	-0.004	0.810
Food allergy	765	105	1698	246	-0.007	0.577	259	23	670	67	-0.014	0.491	506	82	1028	179	-0.011	0.495
Respiratory allergy	535	335	1193	751	0.001	0.935	174	108	448	289	-0.008	0.716	361	227	745	462	0.007	0.673
Other allergies	806	64	1814	130	0.011	0.361	260	22	699	38	0.050	0.016*	546	42	1115	92	-0.011	0.493
Autoimmunity	803	50	1815	94	0.019	0.127	269	8	709	13	0.034	0.103	534	42	1106	81	0.007	0.682
Rheumatoid arthritis	831	22	1880	29	0.033	0.008*	271	6	713	9	0.033	0.117	560	16	1167	20	0.031	**0.052**
Haematological autoimmunity dis.	848	5	1898	11	0.000	0.985	276	1	718	4	-0.012	0.558	572	4	1180	7	0.006	0.723
Thyroiditis	781	72	1789	120	0.038	0.002*	269	8	711	11	0.045	0.034*	512	64	1078	109	0.027	**0.085**
Immunodeficiency	813	40	1824	85	0.006	0.651	272	5	709	13	0.000	0.998	541	35	1115	72	0.001	0.964
Bechterew´s dis.	847	6	1902	7	0.022	**0.088**	275	2	719	3	0.019	0.367	572	4	1183	4	0.023	0.141
Other immunolog. dis.	796	57	1830	79	0.054	0.000*	262	15	707	15	0.088	0.000*	534	42	1123	64	0.037	0.021*
Psoriasis	829	24	1867	42	0.017	0.177	268	9	698	24	-0.002	0.919	561	15	1169	18	0.035	0.027*
Stomach or duodenal ulcer	810	34	1840	62	0.017	0.178	264	11	704	25	0.013	0.544	546	23	1136	37	0.020	0.212
Chronic gastritis	827	17	1845	57	-0.030	0.018*	272	3	714	15	-0.033	0.112	555	14	1131	42	-0.033	0.038*
Liver disease	806	38	1841	61	0.031	0.014*	263	12	713	16	0.058	0.006*	543	26	1128	45	0.016	0.303
Diarrheas	647	197	1496	406	0.027	0.034*	234	41	594	135	-0.041	0.050*	413	156	902	271	0.055	0.001*
Constipation	701	143	1638	264	0.042	0.001*	261	14	689	40	-0.008	0.719	440	129	949	224	0.044	0.006*
Maldigestion, food intolerance	733	111	1646	256	-0.002	0.845	261	14	663	66	-0.065	0.002*	472	97	983	190	0.013	0.405
Malabsoption	822	22	1864	38	0.019	0.137	270	5	723	6	0.043	0.042*	552	17	1141	32	0.006	0.707
Bulimia, anorexia	824	20	1846	56	-0.015	0.247	275	275	729	729	0.000	1.000	549	20	1117	56	-0.027	**0.086**
Flatulence	676	168	1559	343	0.023	**0.076**	235	40	621	108	-0.003	0.875	441	128	938	235	0.029	**0.075**
Weight loss	826	18	1846	56	-0.021	**0.094**	272	3	723	6	0.014	0.521	554	15	1123	50	-0.038	0.017*
Other digestive dis.	817	27	1844	58	0.004	0.764	266	9	710	19	0.018	0.393	551	18	1134	39	-0.005	0.764
Pyrosis reflux	682	162	1555	347	0.010	0.453	224	51	598	131	0.006	0.774	458	111	957	216	0.010	0.519
Gall bladder attack	812	32	1812	90	-0.023	**0.065**	272	3	704	25	-0.064	0.002*	540	29	1108	65	-0.014	0.376
Coeliac disease	834	10	1877	25	-0.005	0.677	275	0	723	6	-0.048	0.024*	559	10	1154	19	0.005	0.762
Hypertensive disease	808	18	1816	48	-0.016	0.222	261	4	689	21	-0.042	0.049*	547	14	1127	27	-0.002	0.910
Ischaemic disease	819	7	1856	8	0.024	**0.067**	264	1	704	6	-0.026	0.229	555	6	1152	2	0.059	0.000*
Other heart dis.	801	25	1791	73	-0.022	**0.091**	255	10	682	28	-0.004	0.855	546	15	1109	45	-0.031	**0.055**
Excessive bleeding	816	10	1848	16	0.016	0.203	263	2	709	1	0.049	0.021*	553	8	1139	15	0.004	0.792
Thrombosis	815	11	1847	17	0.018	0.171	264	1	699	11	-0.048	0.024*	551	10	1148	6	0.060	0.000*
Atrial fibrilation	824	2	1856	8	-0.015	0.254	265	0	706	4	-0.039	**0.065**	559	2	1150	4	0.000	1.000
Arrhythmia, non serious	754	72	1700	164	-0.003	0.821	249	16	657	53	-0.025	0.237	505	56	1043	111	0.003	0.865
Arrythmia, serious	822	4	1856	8	0.002	0.875	264	1	705	5	-0.020	0.358	558	3	1151	3	0.020	0.206
Anemia	712	103	1667	184	0.039	0.002*	257	9	701	11	0.058	0.007*	455	94	966	173	0.021	0.186
High leukocytes level	803	12	1827	24	0.006	0.630	261	5	705	7	0.036	**0.092**	542	7	1122	17	-0.010	0.541
Low leukocytes level	805	10	1831	20	0.007	0.604	262	4	705	7	0.022	0.299	543	6	1126	13	-0.002	0.910
Other problems with leucocytes	812	3	1841	10	-0.012	0.352	266	0	707	5	-0.044	0.039*	546	3	1134	5	0.007	0.679
Other blood diseases	793	22	1804	47	0.004	0.734	260	6	690	22	-0.022	0.295	533	16	1114	25	0.021	0.188
High platelets level	810	5	1844	7	0.016	0.211	264	2	709	3	0.021	0.334	546	3	1135	4	0.014	0.383
Low platalets level	809	6	1829	22	-0.020	0.116	265	1	706	6	-0.025	0.251	544	5	1123	16	-0.021	0.193
Excessive bleeding	768	47	1767	84	0.027	0.038*	263	3	707	5	0.021	0.327	505	44	1060	79	0.019	0.231
Accented blood clotting	806	9	1815	36	-0.029	0.023*	265	1	703	9	-0.039	**0.065**	541	8	1112	27	-0.029	**0.078**
Iron deficiency	706	109	1636	215	0.025	**0.052**	261	5	698	14	-0.003	0.888	445	104	938	201	0.014	0.376
Lymphatic nodes swelling	799	16	1828	23	0.027	0.034*	265	1	707	5	-0.019	0.375	534	15	1121	18	0.038	0.018*
Vitamin B12 deficiency	794	21	1822	29	0.034	0.008*	265	1	706	6	-0.025	0.240	529	20	1116	23	0.048	0.003*
Type1 diabetes	793	6	1805	9	0.015	0.237	259	3	699	3	0.040	**0.060**	534	3	1106	6	0.001	0.939
Crohn's disease	794	5	1808	6	0.020	0.118	261	1	700	2	0.008	0.723	533	4	1108	4	0.025	0.132
Immunodeficiency	785	14	1776	38	-0.011	0.387	258	4	696	6	0.030	0.167	527	10	1080	32	-0.031	**0.058**
Type 2 diabetes	786	13	1787	27	0.002	0.903	259	3	685	17	-0.042	**0.054**	527	10	1102	10	0.036	0.028*
Hypothyroidism	724	75	1674	140	0.028	0.033*	260	2	694	8	-0.016	0.449	464	73	980	132	0.022	0.181
Hyperthyroidism	791	8	1796	18	0.000	0.988	262	0	701	1	-0.020	0.358	529	8	1095	17	-0.002	0.882
Inborn metabolic dis.	795	4	1805	9	0.001	0.963	262	0	698	4	-0.039	**0.069**	533	4	1107	5	0.019	0.254
Obesity	720	79	1630	184	-0.008	0.528	238	24	649	53	0.025	0.241	482	55	981	131	-0.031	**0.062**
Hypoglycemia	794	5	1791	23	-0.028	0.030*	260	2	698	4	0.011	0.594	534	3	1093	19	-0.047	0.004*
Osteoporosis	785	14	1786	28	0.005	0.716	261	1	702	0	0.052	0.015*	524	13	1084	28	-0.009	0.579
Delayed puberty	792	7	1797	17	-0.002	0.859	261	1	692	10	-0.043	0.044*	531	6	1105	7	0.027	0.103
Precocious puberty	793	6	1807	7	0.023	**0.072**	262	0	700	2	-0.028	0.196	531	6	1107	5	0.037	0.024*
Amenorrhea	788	11	1793	21	0.010	0.424	262	262	702	702	0.000	1.000	526	11	1091	21	0.007	0.669
Other metabolic dis.	773	26	1759	55	0.006	0.664	258	4	691	11	-0.001	0.950	515	22	1068	44	0.002	0.909
Melanoma and other skin cancer	828	5	1875	10	0.003	0.793	273	0	715	6	-0.048	0.022*	555	5	1160	4	0.034	0.032*
Breast cancer	831	2	1876	9	-0.019	0.143	273	273	721	721	0.000	1.000	558	2	1155	9	-0.028	**0.084**
Cervix uteri cancer	821	12	1868	17	0.023	**0.067**	273	273	721	721	0.000	1.000	548	12	1147	17	0.023	0.161
Other cancer diseases	824	9	1874	11	0.026	0.042*	273	0	713	8	-0.056	0.009*	551	9	1161	3	0.075	0.000*
Urinary tract infections	606	184	1473	326	0.060	0.000*	245	20	657	45	0.019	0.364	361	164	816	281	0.058	0.000*
Nephrosis, glumerulonephritis	779	11	1778	21	0.009	0.501	264	1	696	6	-0.026	0.234	515	10	1082	15	0.020	0.233
Bladder infection, cystitis	725	65	1666	133	0.014	0.269	261	4	692	10	0.003	0.896	464	61	974	123	0.005	0.782
Prostate hypertrophy	787	3	1773	26	-0.051	0.000*	262	3	676	26	-0.070	0.001*	525	525	1097	1097	0.000	1.000
Gynaecological infections	644	146	1518	281	0.038	0.004*	265	0	700	2	-0.028	0.197	379	146	818	279	0.026	0.116
Cervical precancerosis or cancer	778	12	1774	25	0.005	0.725	265	265	702	702	0.000	1.000	513	12	1072	25	-0.002	0.923
Obstretic complications	760	30	1743	56	0.015	0.262	265	265	702	702	0.000	1.000	495	30	1041	56	0.006	0.737
Recurrent abortions	778	12	1773	26	0.001	0.943	265	265	702	702	0.000	1.000	513	12	1071	26	-0.007	0.659
Kidney stones	778	12	1756	43	-0.030	0.024*	261	4	688	14	-0.017	0.430	517	8	1068	29	-0.037	0.024*
Other genitourinary dis.	771	19	1759	40	0.006	0.669	263	2	682	20	-0.063	0.004*	508	17	1077	20	0.045	0.007*
Glaucoma	798	4	1793	23	-0.036	0.006*	262	2	696	7	-0.011	0.606	536	2	1097	16	-0.050	0.002*
Cataracts, clouding of the lens	793	9	1796	20	-0.002	0.880	260	4	693	10	0.002	0.920	533	5	1103	10	-0.002	0.905
Refractive errors	438	364	982	834	-0.006	0.646	161	103	422	281	-0.009	0.661	277	261	560	553	-0.013	0.436
Hearing loss	773	29	1717	99	-0.041	0.002*	251	13	661	42	-0.021	0.327	522	16	1056	57	-0.051	0.002*
Macular degeneration	791	11	1797	19	0.013	0.319	261	3	694	9	-0.006	0.785	530	8	1103	10	0.024	0.144
Strabismus	786	16	1777	39	-0.006	0.669	258	6	685	18	-0.009	0.687	528	10	1092	21	-0.001	0.930
Sense of smell problems	789	13	1765	51	-0.037	0.005*	258	6	674	29	-0.045	**0.037**	531	7	1091	22	-0.026	0.117
Sense of taste prob.	800	2	1807	9	-0.018	0.167	264	0	699	4	-0.040	**0.065**	536	2	1108	5	-0.006	0.706
Ringing in the ears	741	61	1671	145	-0.010	0.465	239	25	626	77	-0.023	0.285	502	36	1045	68	0.008	0.624
Other sense organs dis.	767	35	1745	71	0.011	0.415	254	10	671	32	-0.016	0.445	513	25	1074	39	0.027	0.100
Sense of motion problems	772	30	1757	59	0.011	0.403	260	4	689	14	-0.016	0.447	512	26	1068	45	0.015	0.355
Amblyopia, lazy eye	768	34	1728	88	-0.014	0.269	256	8	664	39	-0.053	0.014*	512	26	1064	49	0.009	0.595
Extremity neuropathy	802	8	1822	24	-0.015	0.242	265	2	702	9	-0.023	0.281	537	6	1120	15	-0.011	0.499
Multiple sclerosis	805	5	1840	6	0.021	0.108	266	1	710	1	0.023	0.280	539	4	1130	5	0.018	0.261
Epilepsy	805	5	1830	16	-0.013	0.319	264	3	706	5	0.021	0.335	541	2	1124	11	-0.032	**0.052**
Migraine	627	183	1450	396	0.014	0.287	231	36	620	91	0.009	0.676	396	147	830	305	0.003	0.850
Other neurologic dis.	793	17	1812	34	0.008	0.555	263	4	703	8	0.015	0.484	530	13	1109	26	0.001	0.959
Stuttering	799	11	1819	27	-0.003	0.812	263	4	699	12	-0.006	0.764	536	7	1120	15	0.001	0.962
Tics	780	30	1771	75	-0.007	0.601	252	15	685	26	0.045	0.036*	528	15	1086	49	-0.036	0.027*
Muscle twitch, fasciculation	753	57	1705	141	-0.010	0.443	243	24	666	45	0.047	0.029*	510	33	1039	96	-0.041	0.011*
Cramps	746	64	1700	146	0.000	0.971	253	14	663	48	-0.027	0.201	493	50	1037	98	0.010	0.543
Unipolar depressive disorders	765	31	1742	78	-0.009	0.469	252	9	682	18	0.024	0.274	513	22	1060	60	-0.028	**0.084**
Bipolar disorder	786	10	1800	20	0.007	0.587	259	2	691	9	-0.022	0.317	527	8	1109	11	0.023	0.169
Anxiety disorders	745	51	1720	100	0.019	0.136	251	10	674	26	0.003	0.879	494	41	1046	74	0.021	0.200
Alcohol use disorders	787	9	1803	17	0.009	0.509	256	5	687	13	0.002	0.938	531	4	1116	4	0.027	**0.096**
Drug use disorders	788	8	1797	23	-0.011	0.396	258	3	688	12	-0.020	0.347	530	5	1109	11	-0.002	0.901
Post traumatic disorder	785	11	1787	33	-0.016	0.221	259	2	692	8	-0.017	0.437	526	9	1095	25	-0.019	0.239
Obsessive compulsive dis.	783	13	1783	37	-0.012	0.338	257	4	682	18	-0.030	0.158	526	9	1101	19	0.001	0.973
Panic disorder	767	29	1772	48	0.027	0.035*	254	7	690	10	0.043	0.048*	513	22	1082	38	0.017	0.304
Insomnia primary	727	69	1655	165	-0.005	0.685	241	20	658	42	0.030	0.158	486	49	997	123	-0.027	0.103
Learning disability	767	29	1761	59	0.011	0.389	250	11	679	21	0.031	0.156	517	18	1082	38	0.001	0.964
Borderline personality disorder	787	9	1804	16	0.013	0.325	259	2	696	4	0.011	0.608	528	7	1108	12	0.013	0.446
Antisocial personality disorder	787	9	1802	18	0.008	0.554	253	8	691	9	0.061	0.005*	534	1	1111	9	-0.034	0.037*
Attention deficit, hyperactivity	772	24	1779	41	0.024	**0.068**	247	14	685	15	0.084	0.000*	525	10	1094	26	-0.012	0.466
Other mental health dis.	769	27	1777	43	0.030	0.021*	252	9	692	8	0.078	0.000*	517	18	1085	35	0.007	0.691
Erectile dysfunction	783	31	1746	83	-0.021	0.105	235	31	631	82	0.000	0.993	548	0	1115	1	-0.018	0.260
Too low sex appetency	654	160	1473	356	0.001	0.930	231	35	622	91	0.004	0.834	423	125	851	265	-0.013	0.428
Too high sex appetency	749	65	1695	134	0.012	0.368	228	38	612	101	0.002	0.933	521	27	1083	33	0.052	0.001*
Too low sex potency	809	5	1811	18	-0.020	0.123	262	4	697	16	-0.024	0.259	547	1	1114	2	0.000	0.994
Quality of sex	724	90	1628	201	0.000	0.999	244	22	644	69	-0.022	0.304	480	68	984	132	0.007	0.690
Other sexuological dis.	791	23	1762	67	-0.021	0.101	256	10	680	33	-0.018	0.392	535	13	1082	34	-0.021	0.209
Paraphilias (mild)	942	11	2157	20	0.011	0.349	312	5	830	10	0.015	0.433	630	6	1327	10	0.011	0.484
Spondylosis, spondylitis	774	9	1772	18	0.004	0.733	256	2	689	8	-0.017	0.434	518	7	1083	10	0.016	0.338
Backbone pain	517	266	1168	622	-0.010	0.430	184	74	498	199	0.000	0.987	333	192	670	423	-0.025	0.139
Osteoporosis	765	18	1764	26	0.027	0.037*	256	2	696	1	0.050	0.022*	509	16	1068	25	0.017	0.292
Rheumatoid arthritis	758	25	1741	49	0.010	0.467	249	9	673	24	0.000	0.993	509	16	1068	25	0.019	0.258
Scoliosis	646	137	1538	252	0.046	0.000*	229	29	624	73	0.012	0.583	417	108	914	179	0.054	0.001*
Scheuermann's disease	774	9	1762	28	-0.017	0.189	254	4	681	16	-0.024	0.274	520	5	1081	12	-0.007	0.656
Other musculosceletal dis.	758	25	1745	45	0.020	0.127	252	6	680	17	-0.003	0.878	506	19	1065	28	0.031	**0.059**
Osteoarthrosis	742	41	1706	84	0.008	0.555	243	15	666	31	0.027	0.209	499	26	1040	53	-0.004	0.825
Bronchitis	721	62	1634	156	-0.016	0.228	246	12	644	53	-0.054	0.013*	475	50	990	103	-0.002	0.892
Asthma	701	82	1577	213	-0.019	0.139	231	27	626	71	0.005	0.829	470	55	951	142	-0.035	0.035*
Recurrent infections	699	84	1585	205	-0.012	0.345	240	18	637	60	-0.027	0.206	459	66	948	145	-0.013	0.430
Other respiratory dis.	756	27	1734	56	0.007	0.569	248	10	683	14	0.053	0.015*	508	17	1051	42	-0.016	0.330

Numbers of RhD negative subjects without particular disorders, RhD negative subjects with particular disorders, RhD positive subjects without particular disorders, RhD positive subjects with particular disorders, partial Kendall´s Tau and statistical significance, respectively, are shown in six columns of each section. The effect of age on health status was controlled in partial Kendall’s correlation (non-parametric) test. Positive Tau corresponds to a positive association and negative B to a negative association of RhD negativity with incidence of particular disorder. Significant results (p < 0.05) and trends (p < 0.10) are printed in bold. Asterisks indicate results significant in two-sided tests. p values < 0.0005 are coded as 0.000. The effect size is shown as Tau.

**Table 3:** Differences between RhD positive and RhD negative participants in specialised medical doctors the subject had to regularly visit at least once in the past two years.

	RhD-V-	RhD-V+	RhD+V-	RhD+V+	*tau*	*p*
			**All**			
Internal medicine	710	101	1651	213	0.011	0.400
Otolaryngology	720	91	1690	174	0.030	0.021*
Neurology	747	64	1729	135	0.010	0.419
Psychiatry	746	65	1751	113	0.036	0.006*
Gynecology	641	170	1539	325	0.045	0.000*
Surgery	766	45	1740	124	-0.020	0.114
Infectology	797	14	1838	26	0.013	0.318
Orthopedy	705	106	1643	221	0.017	0.188
Dermatology	680	131	1602	262	0.029	0.023*
Other Doctors	610	201	1357	507	-0.025	**0.051**
			**Men**			
Internal medicine	241	30	617	92	-0.028	0.197
Otolaryngology	242	29	646	63	0.028	0.194
Neurology	252	19	669	40	0.025	0.238
Psychiatry	254	17	686	23	0.069	0.001*
Gynecology	270	1	707	2	0.007	0.738
Surgery	249	22	649	60	-0.006	0.792
Infectology	266	5	691	18	-0.020	0.341
Orthopedy	244	27	633	76	-0.012	0.585
Dermatology	240	31	626	83	-0.003	0.872
Other Doctors	223	48	556	153	-0.044	0.040*
		** **	**Women**	** **		
Internal medicine	469	71	1034	121	0.034	0.038*
Otolaryngology	478	62	1044	111	0.031	**0.057**
Neurology	495	45	1060	95	0.001	0.969
Psychiatry	492	48	1065	90	0.017	0.308
Gynecology	371	169	832	323	0.037	0.024*
Surgery	517	23	1091	64	-0.025	0.124
Infectology	531	9	1147	8	0.047	0.004*
Orthopedy	461	79	1010	145	0.030	**0.069**
Dermatology	440	100	976	179	0.042	0.010*
Other Doctors	387	153	801	354	-0.023	0.155

Columns 2–5 show numbers of RhD- or RhD+ subjects that had to (V+) and had not to (V-) visit a doctor of particular specialisation within the past 2 years. Columns 6 and 7 show *Tau* and *P* computed with partial Kendall´s correlation between two binary variables, i.e. the RhD phenotype and the Visiting doctor, controlled for the confounding variable age of a subject. Positivity of *Tau* indicates that RhD negative subjects have had to more frequently visit a doctor of particular specialization. Significant results (*P* < 0.05) and trends (*P* < 0.10) are printed in bold. Asterisks indicate results significant in two-sided tests. Values < 0.0005 are coded as 0.000.

## Discussion

The RhD negative subjects expressed many indices of a worse health status. Men, women or both sexes reported more frequent allergic, digestive, heart, hematological, immunity, mental health and neurological problems. They also reported the usage of more drugs prescribed by doctors per day, attended more specialized doctors, namely, dermatologists, gynecologists, internal medicine doctors, neurologists, and psychiatrists (men) in the past two years, a higher frequency of headaches and being tired more often than RhD positive subjects. Incidence of various diseases and disorders also differed between RhD negative and RhD positive subjects, mostly being higher in the former.

RhD negative subjects have increased the risk of developing of certain heart diseases, respiratory diseases and some immunity and autoimmunity related diseases, for example rheumatoid arthritis. The general pattern suggests that RhD negative subjects could have problems with autoimmunity, could be more resistant to infections of viral origin and could be less resistant to infections of bacterial origin.

The mechanism of the effect of the RhD phenotype on human health status is not clear. RhD protein together with strongly homologous RhCE protein and with also homologous RhAG glycoprotein are all components of a membrane complex of which the function is not quite clear. It is most probably involved in NH_3_ transport and possibly also in CO_2_ transport [18,19]. This complex is associated with spectrin-based cytoskeleton and therefore plays an important role in maintaining the typical shape (biconcave discoid) of human erythrocytes [20]. The biological functions of complexes containing the RhD protein are unknown. However, they might be involved in NH_3_ /NH_4_^+^ detoxification of organs. Ammonia, the product of protein catabolism is extremely toxic, especially for brain cells and must be quickly removed from the sensitive organs. It was observed that the concentration of ammonium is three times higher in red cells than in plasma [20] and it was further suggested that the RhD containing complex plays a key role in its capturing and its transport to the kidneys and the liver [20]. It was also suggested that the complex might participate in intracellular pH regulation [20] and consequently also in the regulation of local oxygen tension. It was suggested that RhD-negativity-associated anoxia in certain parts of the nervous system could be responsible for physiological (and also behavioral) effects of the RhD phenotype [21]. The variation of the oxygen tension in various organs and tissues could, of course, influence also other biological functions, including the functions of the immune system. This could explain why RhD negativity seems to be associated with neurological, mental health and immunological disorders. The probable roles of the RhD-containing complex in keeping the normal morphology and adhesiveness of red cells (for review see [20]) could be responsible for the observed associations of RhD negativity with some haematological and inflammation-related diseases, including arthritis.

Limitations and strength of present study: Using very effective Facebook-based snow-ball method we obtained data from a large number of subjects. However, most of them were relatively young people (mean age was 35.4). Most of the diseases and disorders with the largest public health impact (but possibly not the largest economic impact) start at a higher age in developed European countries such as the Czech and Slovak Republics. This can largely distort the whole picture of the RhD negativity impacts on public health. Future studies (which could be easily done in countries with available national databases of medical records of all citizens) should aim to recruit middle age and senior subjects. Our study compared the health status of RhD negative subjects (16% in general population of Czech and Slovak Republics) with RhD positive subjects, i.e., with the health status of mixed population of RhD positive homozygotes (36% of the general populations within the Czech and Slovak Republics) and heterozygotes (48% the general populations in the Czech and Slovak Republics). The results of the published case-control studies on the effects of the RhD genotype on psychomotor performance [22,23], as well as the heterozygote advantage hypothesis, however, suggest that the health status of RhD positive homozygotes and heterozygotes differs. In further studies concentrated on particular disorders, smaller populations of subjects should be RhD genotyped using molecular biology techniques and then the health status of all three RhD genotypes have to be compared. In the present study, the health status data were collected using a questionnaire. This enabled to study of the effects of the RhD phenotypes on rarer disorders using a large population sample. Of course, more precise and more detailed data could be obtained from medical records. Primarily, we have run the study to confirm or disprove the alarming results of a previous small scale studies performed on non-typical populations. However, we had no *a priory* hypotheses which health-related variables should correlate with RhD phenotype or which disorders should occur more frequently in RhD negative subjects. Therefore, the present study had a more or less explorative character. Hence, we have reported the results of statistical tests without formal correction to multiple tests. It should be noted, however, that, for example, we have obtained 41% positive results for the ordinal health status variables and 20% positive results for binary health status variables. Theoretically, only 5% of false positive results should be expected in multiple tests. The main strength of the present study is the absence of any sieve effect, which could result in publication bias in other types of studies. Positive results of particularly observational or experimental studies and partly also meta-analytic studies, could be an artefact of intentional or unintentional “cherry-picking”; i.e. preferential or even exclusive publication of positive results. In our study we have searched for the effects of the RhD phenotype on all diseases and all disorders having high enough incidences in the Czech population (n = 154) and we have reported all, both positive and negative results.

## Conclusions

Some of the associations observed the present study were relatively strong and some of them concerned rather frequent disorders. Therefore, the total impact of frequency of RhD negative homozygotes in the general population on public health could be large.

The aim of the present study was to search for indices of validity of the heterozygote advantage hypothesis, namely for the indices of impaired health status of RhD negative subjects. It must be reminded, however, that the observed specific disease burden of the RhD negative subpopulation is in an agreement with predictions of this hypothesis but does not prove its validity. The higher disease burden in RhD negative homozygotes could be compensated either by increased fitness of heterozygotes (heterozygote advantage hypothesis) or by still unknown selection pressure in favor of RhD negative subjects. In this context, the shorter reaction times of RhD negative, *Toxoplasma*-free blood donors [7] and university students [8] and higher intelligence in RhD negative, *Toxoplasma*-infected soldiers [11] should be remembered. It could be speculated to what extent the highly uneven distributions of RHD minus alleles in world populations might be the result of a founder event and a gene flow [24] and to what extent it is also modulated by specific selection pressures caused by differences in the geographical distribution of a disease or diseases.

## Supporting Information

S1 File Excel file containing the data set. (XLSX)

S2 File Excel sheet for computing the partial Kendall correlation test. (XLS)
